# Thymic Amyloidosis Mimicking Thymoma: A Case Report

**DOI:** 10.70352/scrj.cr.25-0196

**Published:** 2025-08-19

**Authors:** Asuka Tomida, Motoki Yano, Tomohiro Setogawa, Ryotaro Katsuya, Chihiro Furuta, Naoki Ozeki, Takayuki Fukui

**Affiliations:** Division of Chest Surgery, Department of Surgery, Aichi Medical University, Nagakute, Aichi, Japan

**Keywords:** amyloidosis, mediastinal tumor, thymoma

## Abstract

**INTRODUCTION:**

The most common anterior mediastinal tumors are thymic epithelial tumors, including thymoma, and other kinds of diseases are relatively rare. Here, we report our experience in treating a patient with amyloidosis of the thymus, a very rare type of mediastinal lesion.

**CASE PRESENTATION:**

We experienced thymic amyloidosis mimicking a thymoma. A 66-year-old male underwent thymectomy for an anterior mediastinal tumor, which was incidentally pointed out with chest CT. The pathological examination revealed that eosinophilic unstructured substances were present around the atrophied thymic tissue, suggesting the presence of amyloid deposits. Thus, the postoperative pathological diagnosis was thymic amyloidosis. Thymic amyloidosis is extremely rare. Since other amyloid deposits were identified in the gastric mucosa of this patient, he was diagnosed with systemic amyloidosis, and chemotherapy was initiated. The patient had no progression of amyloidosis for 21 months after surgery.

**CONCLUSIONS:**

To the best of our knowledge, only 8 cases of thymic amyloidosis, including the present case, have been reviewed in the literature. Although thymic amyloidosis is extremely rare, an unfavorable prognosis has occasionally been reported. In addition, thymoma and thymic amyloidosis have similar imaging findings, thus making it difficult to distinguish between them. Therefore, thoracic surgeons may need to be aware that thymic amyloidosis is included in the list of anterior mediastinal lesions.

## Abbreviations

AChR
: acetylcholine receptor
AL
: amyloid light chain
MG
: myasthenia gravis

## INTRODUCTION

Noninvasive anterior mediastinal lesions are usually asymptomatic and incidentally found. Among them, thymoma is recognized as the most common histology, accounting for 40% of all mediastinal lesions resected in Japan during 2021.^1)^ Subsequently, thymic carcinomas, teratomas, and various thymic cysts are also treated as rare anterior mediastinal lesions. Of the anterior mediastinal lesions suspected to be thymomas on radiographic studies, few patients had benign, nonneoplastic lesions. However, in clinical practice, understanding the presence of such lesions is also important. Here, we report our experience of surgically treating a very rare case of thymic amyloidosis mimicking thymoma.

## CASE PRESENTATION

**Fig. 1:**
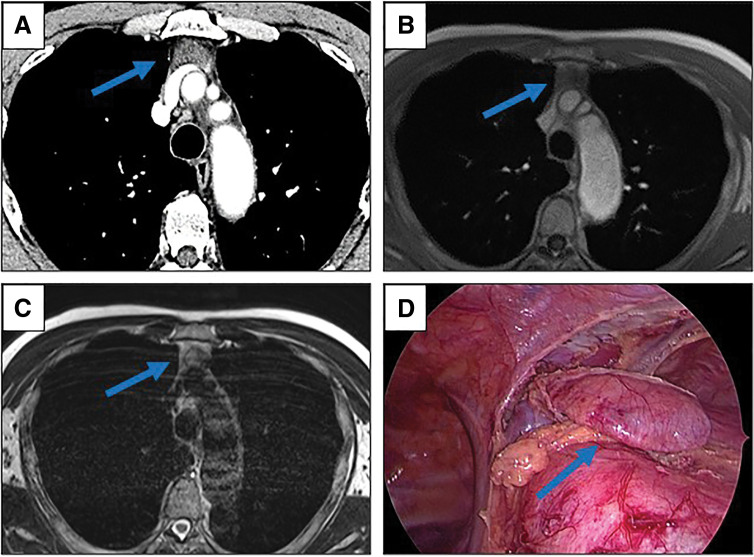
(**A**) Chest contrast-enhanced CT revealed an anterior mediastinal lesion with a maximum diameter of 5 cm. The lesion was slightly enhanced homogeneously (blue arrow). MRI revealed low signal intensity on T1-weighted images (blue arrow) (**B**) and iso-intensity on T2-weighted images (blue arrow) (**C**). (**D**) Intraoperative view. The normal thymic tissue appeared abundant for his age. The lesion did not invade the surrounding organs and was demarcated from the normal thymic tissue (blue arrow).

A 66-year-old male with a diagnosis of left cerebellar hemorrhage was admitted to our hospital. An anterior mediastinal lesion was incidentally pointed out with a chest CT. Subsequently, he was referred to our department. The patient’s medical history included hypertension, benign prostatic hypertrophy, chronic obstructive pulmonary disease, acute epiglottitis, and inguinal hernia. No symptoms, including myasthenia gravis (MG), were observed. The serum anti-acetylcholine receptor (AChR) antibody level was low (<0.2 nmol/L). Radiographically, contrast-enhanced CT revealed an anterior mediastinal lesion with a maximum diameter of 5 cm, which was slightly enhanced homogeneously (**Fig. 1A**). MRI revealed low signal intensity on T1-weighted images and iso-intensity on T2-weighted images, and a simple thymic cyst was excluded from the differential diagnosis (**Fig. 1B** and **1C**). According to the above preoperative evaluation, we suspected that this lesion was a thymoma. After the patient’s general condition had recovered from the brain hemorrhage, he underwent thoracoscopic thymectomy. The lesion did not invade the surrounding organs and was demarcated from the normal thymic tissue, although the normal thymic tissue appeared abundant for his age (**Fig. 1D**). The operative time was 68 min, and the amount of blood loss was 10 g. The patient’s postoperative course was uneventful, and he was discharged on the fourth postoperative day.

**Fig. 2:**
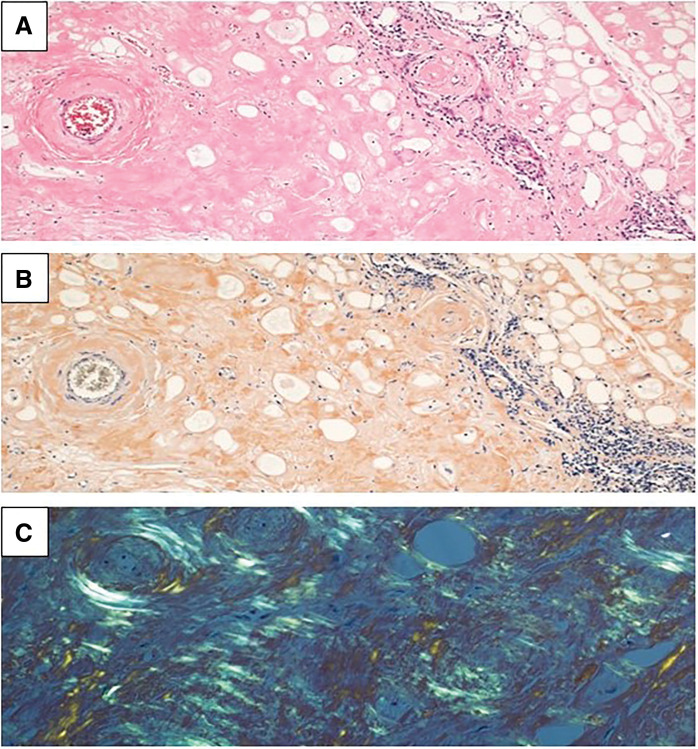
Hematoxylin and eosin staining revealed deposition of eosinophilic unstructured substances around the atrophied thymic tissue (**A**) (×100). The unstructured substance was stained reddish-brown with direct fast scarlet staining (**B**) (×100), which revealed apple-green color under polarized light microscope (**C**) (×100). These findings suggest the presence of amyloid.

The pathological examination revealed that eosinophilic unstructured substances were present around the atrophied thymic tissue. The unstructured substance stained a reddish-brown color with direct fast scarlet staining, which suggested the presence of amyloid (**Fig. 2A** and **2B**). Polarized light microscopy revealed an apple-green color of the amyloid (**Fig. 2C**). Based on these findings, the anterior mediastinal lesion was diagnosed as a thymic amyloidosis. Additional whole-body examinations were performed to check for amyloid deposits in other organs. We conducted echocardiography, scintigraphy, and MRI of the heart, neurological examination, and skin biopsy, but these examinations showed no evidence of amyloid deposition. While we did not conduct a renal biopsy, amyloid deposits were found in the gastric mucosa by upper gastrointestinal endoscopy. In addition, since immunoglobulin M-λ was detected by electrophoresis, he was diagnosed with amyloid light chain (AL) amyloidosis. Subsequently, daratumumab in combination with cyclophosphamide, bortezomib, and dexamethasone was initiated in the department of hematology. Twenty-one months after surgery, he is followed up every 3–4 months and has shown no significant changes in amyloidosis.

## DISCUSSION

We treated a mediastinal tumor and found that the patient had systemic amyloidosis, whose initial presentation was an anterior mediastinal tumor. Localized amyloidosis accounts for 13% of total amyloidosis.^2)^ In localized disease, deposits are limited to a single organ or tissue. In systemic disease, amyloid deposits are found in various organs, such as the heart, respiratory tract, gastrointestinal tract, including tongue and lip, skin, nail, and bladder. The prognosis of systemic amyloidosis depends on the affected organs. Especially in cases with cardiomyopathy due to cardiac amyloidosis, a worse prognosis has been reported.

To the best of our knowledge, only 8 cases of thymic amyloidosis, including the present case, have been reviewed in the literature (**Table 1**).^3–9)^ The average age of the patients was 59 years, and the female gender was predominant. The mean maximal tumor diameter was 6.7 cm. Surgical extirpation was performed in all but one patient. Among them, 3 patients with symptoms and/or a diagnosis of MG have been reported. All but one of the patients with MG were diagnosed with AL amyloidosis. Interestingly, thymic amyloidosis leads to the development of MG.^5,7)^ Generally, thymic hyperplasia and thymoma often induce MG. However, the mechanisms by which thymic amyloidosis induces MG are not clear. Son et al.^5)^ reported a case of thymic amyloidosis and MG and hypothesized that amyloidosis was provoked by the deposition of anti-AChR Ab-derived misfolded light chains. Chapman et al.^7)^ reported that thymic calcification was apparent in cases combined with thymic amyloidosis and MG. Thymic calcification is frequently found in cases of thymic amyloidosis. Similarly, thymic calcification is common in thymomas. Features of CT findings of amyloidoma in various organs include low-density areas consistent with amyloid deposition and poor enhancement due to decreased blood flow caused by infiltration of amyloid into the vessel wall and organ parenchyma.^10)^ Furthermore, organomegaly or wall thickening is also a feature of CT findings.^10)^ However, in this case, we first suspected a thymoma because the lesion had a nodular contrast effect. In retrospect, it is possible that the area of amyloid deposition may have had a partial lack of contrast effect. Therefore, it is not easy to distinguish between thymomas and thymic amyloidosis based on their CT findings. In fact, cases of thymic amyloidosis mimicking thymoma have been reported previously. In recent years, minimally invasive thymectomies using thoracoscopic or robotic techniques have become available. Regardless of the disease, both diagnostic and therapeutic thymectomy may be recommended.

**Table 1:** Reported cases of thymic amyloidosis

#	Age	Gender	MG	Tumor size (cm)	Calcification	Lymphoplasma cell infiltration	Amyloid type	Treatment	Prognosis
1	33	Female	(−)	8	(+)	(−)	AA(+)	Surgical resection	NA
2	55	Female	(−)	7	(+)	(+)	AL(+)	Surgical resection	NA
3	46	Female	(+)	4	(+)	(+)	AL(+)	Cholinesterase inhibitor therapy, Immunoglobulin therapy, Steroid therapy, Surgical resection	3 months
4	85	Male	(+)	4	(+)	NA	AL(+)	Surgical resection	15 months
5	45	Female	(+)	8	(+)	NA	AL(−)	Surgical resection	11 months
							AA(−)	Steroid therapy	
6	78	Female	(+)	8	(−)	(−)	AL(+)	Steroid therapy	NA
							AA(−)		
7	71	Female	(−)	10	(+)	NA	AL(+)	Surgical resection	3 years
8 (present case)	66	Male	(−)	4	(−)	(−)	AL(+)	Surgical resection	21 months

AA, amyloid A protein; AL, amyloid light chain; MG, myasthenia gravis; NA, not available

## CONCLUSIONS

We experienced thymic amyloidosis mimicking a thymoma. Although thymic amyloidosis is extremely rare, an unfavorable prognosis has occasionally been reported in the systemic type. Thus, if thymic amyloidosis is detected, it is essential that a subsequent systemic search be performed. Therefore, thoracic surgeons may need to be aware that thymic amyloidosis is included in the list of anterior mediastinal lesions.

## DECLARATIONS

### Funding

No funding was received for this case report.

### Authors’ contributions

AT wrote the manuscript.

TF reviewed and revised the manuscript.

MY performed the surgery.

All authors read and approved the final manuscript and agree to be held accountable for all aspects of this case report.

### Availability of data and materials

The datasets supporting the conclusions of this article are included within the article.

### Ethics approval and consent to participate

This case report was approved by the Ethics Committee of the Aichi Medical University School of Medicine (Approval No. 2024-226). Written informed consent was obtained from the patient for participation.

### Consent for publication

Written informed consent was obtained from the patient for publication of this case report and accompanying images.

### Competing interests

The authors declare that they have no competing interests.

## References

[1] Yoshimura N Sato Y Takeuchi H Thoracic and cardiovascular surgeries in Japan during 2021: Annual report by the Japanese Association for Thoracic Surgery. Gen Thorac Cardiovasc Surg 2024; 72: 254–91. 38421591 10.1007/s11748-023-01997-6 PMC10955033

[2] Charlot M Seldin DC O’hara C Localized amyloidosis of the breast: a case series. Amyloid 2011; 18: 72–5. 21501022 10.3109/13506129.2011.570817

[3] Takamori S Yano H Hayashi A Amyloid tumor in the anterior mediastinum: report of a case. Surg Today 2004; 34: 518–20. 15170548 10.1007/s00595-004-2750-4

[4] Ha SY Lee JJ Park H Localized primary thymic amyloidosis presenting as a mediastinal mass: a case report. J Pathol Transl Med 2011; 45(Suppl 1): S41–4.

[5] Son SM Lee YM Kim SW Localized thymic amyloidosis presenting with myasthenia gravis: case report. J Korean Med Sci 2014; 29: 145–8. 24431920 10.3346/jkms.2014.29.1.145 PMC3890467

[6] Sato F Hata Y Otsuka H Isolated nodular thymic amyloidosis associated with diplopia. Ann Thorac Surg 2014; 98: 1470–2. 25282219 10.1016/j.athoracsur.2013.11.084

[7] Chapman KO Beneck DM Dinkin MJ. Ocular myasthenia gravis associated with thymic amyloidosis. J Neuroophthalmol 2016; 36: 50–2. 25822660 10.1097/WNO.0000000000000241

[8] Kato Y Okuda M Fukuda K Sclerosing thymoma-like thymic amyloidoma with nephrotic syndrome: a case report. J Med Case Rep 2017; 11: 216. 28877738 10.1186/s13256-017-1370-8 PMC5588697

[9] Ikeda S Arai H Sekine A Anterior mediastinal amyloidosis mimics thymic carcinoma. JTO Clin Res Rep 2020; 1: 100043. 34589940 10.1016/j.jtocrr.2020.100043 PMC8474463

[10] Ohta S Tomozawa Y Watanabe S Update on imaging diagnosis of amyloidosis, abdomen: CT・MRI (Amiroido-sisu no gazousindan update, Hukubu: CT・MRI). Gakken Medical Shujunsha Co. Ltd. 2012; 32:1155–60.

